# Developing CIRdb as a catalog of natural genetic variation in the Canary Islanders

**DOI:** 10.1038/s41598-022-20442-x

**Published:** 2022-09-27

**Authors:** Ana Díaz-de Usera, Luis A. Rubio-Rodríguez, Adrián Muñoz-Barrera, Jose M. Lorenzo-Salazar, Beatriz Guillen-Guio, David Jáspez, Almudena Corrales, Antonio Íñigo-Campos, Víctor García-Olivares, María Del Cristo Rodríguez Pérez, Itahisa Marcelino-Rodríguez, Antonio Cabrera de León, Rafaela González-Montelongo, Carlos Flores

**Affiliations:** 1 Genomics Division, Instituto Tecnológico y de Energías Renovables (ITER), 38600 Santa Cruz de Tenerife, Spain; 2 Research Unit, Hospital Universitario Nuestra Señora de Candelaria, 38010 Santa Cruz de Tenerife, Spain; 3 Área de Medicina Preventiva y Salud Pública, Universidad de La Laguna, 38010 Santa Cruz de Tenerife, Spain; 4 CIBER de Enfermedades Respiratorias, Instituto de Salud Carlos III, 28029 Madrid, Spain; 5 Facultad de Ciencias de La Salud, Universidad Fernando Pessoa Canarias, 35450 Las Palmas de Gran Canaria, Spain

**Keywords:** Genotype, Population genetics

## Abstract

The current inhabitants of the Canary Islands have a unique genetic makeup in the European diversity landscape due to the existence of African footprints from recent admixture events, especially of North African components (> 20%). The underrepresentation of non-Europeans in genetic studies and the sizable North African ancestry, which is nearly absent from all existing catalogs of worldwide genetic diversity, justify the need to develop CIRdb, a population-specific reference catalog of natural genetic variation in the Canary Islanders. Based on array genotyping of the selected unrelated donors and comparisons against available datasets from European, sub-Saharan, and North African populations, we illustrate the intermediate genetic differentiation of Canary Islanders between Europeans and North Africans and the existence of within-population differences that are likely driven by genetic isolation. Here we describe the overall design and the methods that are being implemented to further develop CIRdb. This resource will help to strengthen the implementation of Precision Medicine in this population by contributing to increase the diversity in genetic studies. Among others, this will translate into improved ability to fine map disease genes and simplify the identification of causal variants and estimate the prevalence of unattended Mendelian diseases.

## Introduction

The Canary Islands are a Spanish archipelago of seven main islands located in the Atlantic Ocean, a hundred kilometers off the Northwest African coast, with Cape Juby (Morocco) being the closest mainland point. Before the XV century, when the archipelago was fully incorporated into the European world, it was inhabited by aborigines^1^ with their most likely origin in the Berber population from North Africa^2,3^. This and subsequent historic events, including the European colonization of diverse origins and the slave trade from western sub-Saharan African populations^4^, have shaped the genetic makeup of Canary Islanders, as has been established by the historical burial remains and the diverse ancient DNA studies^5–7^. Early genetic studies have supported that the current Canary Islanders could be modeled as descendants of a recent three-way admixture event. Note, however, that these studies just considered the major continental populations and did not assess the substructure components in the parental populations. Sexual asymmetry in the admixture event has been invoked to explain the observed unbalanced proportions of indigenous parental lineages from the non-recombining portion of the Y chromosome (NRY) and the mitochondrial DNA (mtDNA) in current inhabitants^8^. This has been explained by a steady increase of European male lineages soon after the conquest, whereas the indigenous founder mtDNA lineages have remained at roughly constant frequencies until the present days^9,10^. Overall, while there is a large interindividual variability in the ancestry proportions in the current inhabitants, they have been estimated to an average of 75–83% European (EUR), 17–23% North African (NAF), and 3% or less sub-Saharan Africa (SSA)^11,12^. The most recent analyses based on genome-wide single nucleotide polymorphism (SNP) array data evidenced that these numbers can be as high as up to 29.9% NAF and 9.2% SSA ancestries in some individuals^13^. Most importantly, they have also evidenced broad genomic regions of Canary Islanders that tend to concentrate African alleles and that are enriched in genes involved in diverse complex diseases, which is highly suggestive of characteristic footprints of local adaptations.

The success of Next Generation DNA Sequencing (NGS) represents a milestone in how genetic variation is discovered and analyzed nowadays. It has opened new horizons to improve disease diagnosis, prognosis, and treatment, and constitutes a central element of the Precision Medicine paradigm^14^. Nevertheless, the genetic knowledge of human traits and diseases remain to be almost entirely based on results from studies in European populations^15,16^. This results in an underrepresentation of ethnic diversity and a conspicuous lack of accuracy in the understanding of the genetic architecture and the biology of human traits, thus challenging the translation of this knowledge into generalizable clinical applications^17–19^. While there are differences in allele frequencies across populations^20^, the rarer the variant, the more likely for it to be more locally circumscribed to populations^21^. The estimations indicate that while the proportion of rare variants (minor allele frequency [MAF] < 0.5%) shared among populations from the same continent is 70–80%, the proportion of shared rare variants drops down to 10–30% among populations from different continents, and are, therefore, poorly represented in the reference catalogs of genetic variation^22,23^. Most importantly, deleteriousness is known to accumulate on the lower end of the allele frequency spectrum^24^. The application of NGS, both through whole-exome sequencing (WES) and whole-genome sequencing (WGS), has drastically increased the diagnostic yield in patients of European ancestry, such as for autosomal dominant retinitis pigmentosa^25^, severe intellectual disability^26^, and Mendelian conditions in a broad sense^27,28^. Because of that, the substantial benefits of incorporating participants from diverse ancestries and recently admixed populations to improve the discovery of disease genes have been evidenced^15^. This highlights the urgent need for building local population reference catalogs of genetic diversity to efficiently facilitate the identification of disease genes^29^. Developing these population catalogs of genetic variation is such of importance that multiple countries have made huge efforts to develop their own based on the study of a representative control strata of the populations while preserving genetic diversity specificities, many of which have been recently integrated in the Genome Aggregation Database (gnomAD)^30^. Iran^31^, Japan^32^, Korea^33^, Finland^34^, Spain^35^, the United Kingdom^36^, or the Netherlands^37^ are some of the countries which have seen the necessity to develop their own catalogs of genetic variation. The availability of population-specific catalogs of variation allows to identify genetic peculiarities of the population^38^ as is key for identifying disease-causing variants in both rare diseases and complex human traits^32,36^. This has been recently exemplified by the striking detection of recessive deficiencies in two genes of the type I interferon pathway, critically involved in life-threatening viral diseases including COVID-19, at relatively high frequency (> 1%) in Polynesia and Inuits, while these deficient are extremely rare or absent from other regions of the world^39,40^. For the particular case of the Canary Islands, one of the early examples of its benefit has been recently demonstrated to support the underdiagnosis of Wilson disease, a rare difficult-to-diagnose disease^41^. The benefits have been also shown for complex traits, which is more evident for the case of isolated populations, as the cases of the Northern Greek populations of the Pomak villages and the Mylopotamos villages in Crete^42,43^, or the population of Cilento from Southern Italy^44^, highlighting for example the increase in allele frequency of variants involved in haematological traits, among others. Besides these applications, there are substantial gains of incorporating information from the population of interest to reference panels during variant imputation, as it improves the power to identify disease variants and enables fine-mapping in genome-wide association studies of complex traits^18,42,43,45^. Thus, developing a population-specific catalog of genetic variation is an essential step to optimally develop generalizable clinical applications.

The historical conquest and admixture events, jointly with the isolation and inbreeding, as well as the likely local adaptation processes, have shaped the current genetic background of the Canary Islands population, constituting the population with the largest proportion of North African ancestry among Southwestern Europeans^12,13^. Despite the increase in awareness of the necessity of including more diversity in the genetic studies^46,47^, the currently available catalogs of genetic variation have a strong bias towards the representation of northern, western, and central European populations. In particular, it has been shown that the African genomic ancestry has important biomedical implications for European populations^12^ and it has been associated with risk in cardiovascular, renal, and respiratory diseases, as well as in diabetes, among others^48–51^. Strikingly, for some of them, the estimates of prevalence and/or their complications are higher in the Canary Islands than in other mainland regions of Spain. This is the case of asthma and allergic diseases in children^52,53^, and of diabetes, obesity, and hypertension in all age groups^53^. Besides, not only the morbidity but also the mortality due to diabetes is increased, being three-fold higher among Canary Islands compared to the rest of the Spanish populations^54^. Despite the interest considering that the Canary Islanders exhibit the largest proportion of NAF ancestry known to date in the European diversity landscape^13^, there is a lack of data from North African populations on the public catalogs representing human genetic diversity^55^, not even being covered by the African Genome Variation Project^46^.

Here we aimed to establish the foundations to develop a reference genetic catalog of the Canary Islands population (termed CIRdb). Providing an unbiased catalog of natural genetic variation of this population, preserving unique genetic African ancestry footprints, is a first necessary resource for optimal development of Precision Medicine in this population^56^.

## Materials and methods

### Study samples and genotyping

The study was approved by the Research Ethics Committee of the Hospital Universitario Nuestra Señora de Candelaria and performed according to The Code of Ethics of the World Medical Association (Declaration of Helsinki).

The samples were obtained from the cohort study ‘CDC of the Canary Islands’^57^, which constitutes the most extensive general population cohort for epidemiological studies of the Canary Islands archipelago. Briefly, this cohort involves health survey data and samples from nearly 7000 randomly selected donors providing informed consent through personal interviews, aged between 18 and 75 years from the seven main islands and without gender bias. Despite it is well-known to properly represent the Islands’ population^58^, the CDC cohort lacks deep genetic assessment despite the recognized necessity^53^. A subset of 416 individuals from the cohort was previously assessed to characterize inbreeding, selection, and the mosaic nature of the Canarian genomes^13^. We nested the current study in that cohort, particularly focusing on a subset of 1024 donors (483 males and 541 females), fulfilling that they self-reported absence of cardiovascular, metabolic, immunologic, or cancer diseases, and that the four grandparents were born on the same island. The latter criterion was relaxed to accommodate donors from Fuerteventura, where the selection imposed self-reporting three grandparents born on the island. The samples were pseudonymized for the purposes of this study.

DNA was extracted from peripheral blood using the Blood genomicPrep Mini Spin Kit (Cytiva, Marlborough, MA) following the manufacturer’s recommendations. We relied on the Axiom® Genome-Wide Human CEU 1 Array (Affymetrix, Santa Clara, CA) to obtain genotypes from 587,352 variants with the support of the National Genotyping Center (CeGen), Universidad de Santiago de Compostela Node. The AffyPipe v2.10.0 open-source pipeline^59^ was used to process the image files and to run the first genotyping quality controls (QCs) based on a Dish-QC (i.e. an own statistic from the tool which allows to evaluate the signal of non-polymorphic positions) with values higher than 0.82, and samples with call rate (CR) above 0.93. Additionally, after running AffyPipe, more SNP QCs were implemented to select accurate variants by means of SNPolisher package in R 3.2.2 environment^60^ and according to next parameters which were extracted from ‘ps.performance.txt’ file: Fisher’s Linear Discriminant (FLD) > 4.375, Heterozygous Strength Offset (HetSO) ≥  − 0.5, SNP CR ≥ 95, and variants with assigned rsID. Next, the R environment and PLINK v1.07^61^ were used for additional standard QC steps, including the identification of variants in non-autosomal chromosomes, and variants with large deviations from Hardy–Weinberg equilibrium (*p* < 1.0 × 10^−6^), large missingness rate (CR < 0.95), or MAF < 0.01, and the identification of samples with gender discordances (self-declared *vs.* genetically inferred), outlier heterozygosity rate, and family relationships with other study donors (PIHAT > 0.2). For the purpose of this particular study, we only considered the donors declaring four grandparents born on the same island (three grandparents in the context of Fuerteventura). After variant and sample QCs, the dataset was ready for ulterior population analysis.

### Reference datasets and analyses

The genetic background of the Canary Islanders was evaluated under two different scenarios: one focusing only the Canary Islanders, and another comparing the Canary Islanders against reference populations. Each scenario involved a different number of samples and variants (based on the use of different filters) (Table 1).

**Table 1:** Summary of the population analyses with indications of the number of samples and SNPs involved.

Analysis	Samples		Filters involved in SNP selection	#SNPs used
	Canary Islanders	Reference population		
PCA	863	522	LD SNPs (r^2^ > 0.5) and regions^a^	101,271
	863	–	LD SNPs (r^2^ > 0.15) and regions^a^	116,959
	617	–	LD SNPs (r^2^ > 0.15) and regions^a^	116,959
ADMIXTURE	690	522*	LD SNPs (r^2^ > 0.5) and regions^a^	101,271
ELAI	690	522	Not pruned	114,929

PCA, Principal Component Analysis; LD, linkage disequilibrium. ^a^ Regions of long-range linkage disequilibrium were defined elsewhere13. *As a sanity check, some analysis included other European populations from the south of the continent (i.e., Toscani in Italy [TSI, *N* = 106] and Iberian Populations in Spain [IBS, *N* = 106] from 1KGP).

#### Reference population datasets

To place the genetic variation of Canary Islanders in context, we accessed reference data from The 1000 Genomes Project (1KGP) Phase 3^62^ for EUR and SSA populations. Europeans included data from Finnish in Finland (FIN) (*N* = 99), British in England and Scotland (GBR) (*N* = 91), and Utah Residents with Northern and Western European ancestry (CEU) (*N* = 99). Note, however, that for specific analysis (i.e., for ADMIXTURE), we also included other European populations from the south of the continent (i.e., Toscani in Italy [TSI, *N* = 106] and Iberian Populations in Spain [IBS, *N* = 106] from 1KGP). Given that using alternative African populations from 1KGP or smaller subsets of individuals from the parental populations provide equivalent admixture results in Canary Islanders^13^, we used only the Yoruba population in Ibadan (Nigeria) (YRI) (*N* = 108) as representatives of the SSA populations. NAF populations were represented by the 125-individual dataset that is publicly available and genotyped using Genome-Wide Human SNP Array 6.0 (Affymetrix) for 732,532 variants^63^. The intersection of the reference datasets (*N* = 522) with those of Canary Islanders (*N* = 863) was implemented using ‘–bmerge’ command on PLINK v1.9^64^ to merge into one file including only the variants that were shared among populations. This process left us with data from 1385 individuals and 114,929 variants as the final filtered dataset for the downstream analyses involving both Canary Islanders and the reference population datasets.

#### Principal component analysis

Principal Component Analysis (PCA) among Canary Islanders and reference population datasets (*N* = 1385) was computed with PLINK based on a dataset of 101,271 variants, which excluded variants in high linkage disequilibrium (LD) and those that were located in regions of long-range LD as performed elsewhere^13^. For the comparisons within the Canary Islands populations, a pairwise *r*^2^ threshold of 0.15 was used to maintain 116,959 variants for the analyses in the 863 individuals collected from the archipelago.

#### Ancestry inferences

Two approaches were conducted to assess the genetic ancestry partitions of the subjects under study: 1) a direct global ancestry estimation using ADMIXTURE v1.3.0^65^; and 2) a global ancestry estimation from local ancestry inference for admixed individuals by means of ELAI v1.01^66^. We have shown that, compared to other local ancestry estimators, ELAI offers the least biased estimates for this population^13,67^. Note that ancestry estimations inferred for the reference populations (i.e., Europeans and North Africans) should be considered with caution due to the existence of genetic drift effects that have not been properly modelled^13^. Nevertheless, this does not affect the ancestry inferences obtained for the Canary Islanders.

ADMIXTURE implements a maximum likelihood estimation to calculate the individual ancestries averaged across the genome. For this approach, 2 to 7 ancestral populations (K) and 10-times cross-validation were tested to estimate the best fitting K. In order to avoid spurious clusters in the ADMIXTURE results due to the existence of inbreeding, which we have evidenced in the populations from smaller islands^13^, we further pruned the Canary Islands dataset to be considered for this particular analysis. For that, we calculated the runs of homozygosity (ROHs) with PLINK following a sliding window approach^68^. We allowed that a minimum window density of 50 kb/SNP was asserted, as well as one heterozygous variant and up to five missing calls per window. For tracts with a minimum length of 500 kb, 50 was the minimum number of homozygous SNPs to consider a tract as a ROH, and 100 kb was the maximum gap allowed between two consecutive SNPs to include them in the same ROH. The hit rate of all scanning windows containing a variant must be at least 0.05 to comprise a certain ROH. Finally, the samples with ROH lengths above the 80th percentile (1.08 Mb) were excluded from the analysis. This left us with 690 Canary Islanders for this particular analysis, providing a final dataset of 1212 samples (including 552 individuals from the reference populations) and the 101,271 pruned variants which have been previously used for the PCA. Additionally, some European populations from the south of the continent (TSI and IBS from 1KGP) were included into the ADMIXTURE analysis as a sanity check.

For local ancestry estimation, ELAI uses a two-layer hidden Markov model to assess the local ancestry in the individuals. The same subset of 690 Canary Islanders was evaluated to match the results from both ancestry inferences approaches. Based on our previous observations, we assumed a three-way admixture model of EUR, NAF, and SSA to calculate the structure of local haplotypes given that both approaches (i.e., ADMIXTURE and ELAI) provided similar estimates^13^. Moreover, 14 generations since the last admixture event was assumed based on our previous findings^13^. We excluded SNPs with MAF < 0.01 or with missing position information in any of the reference datasets. Subsequently, the global ancestry estimation was calculated considering the average of each ancestry per individual per chromosome and summarizing all the information into a unique value per individual per ancestry. Therefore, global inferences based on ELAI algorithm were implemented using 1212 individuals (690 Canary Islanders and 522 individuals from reference datasets) and 114,929 variants (given that independence of SNPs is not a requirement for this approach).

## Results

### Samples included in the CIRdb catalog

**Figure 1:**
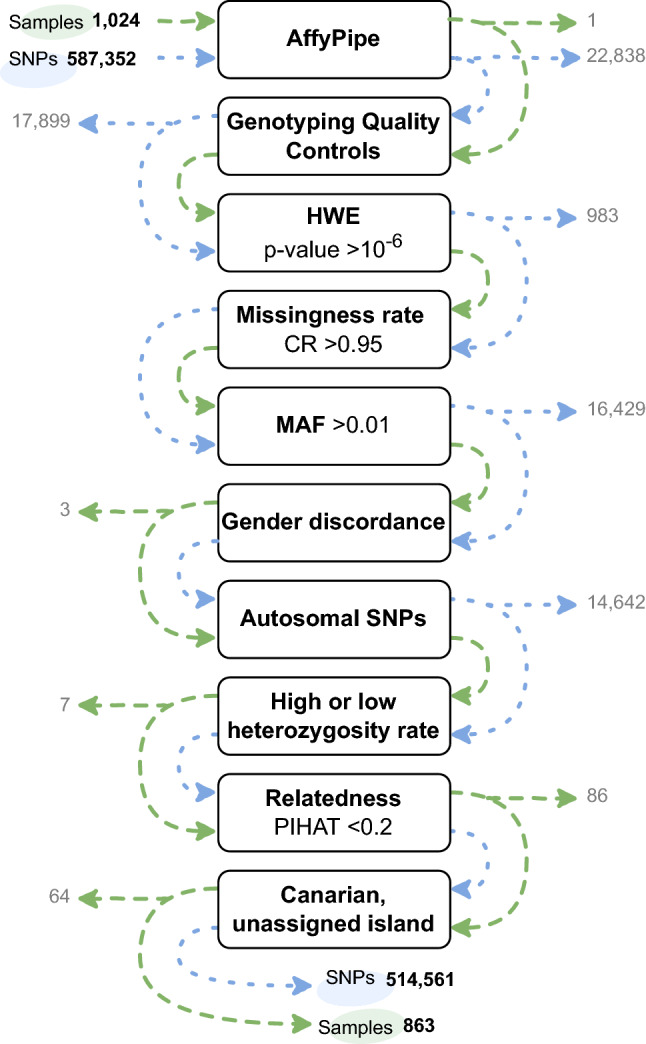
Schematic representation of the variants (blue) and samples (green) that were filtered out based on quality control steps. SNPs, single nucleotide polymorphisms; HWE, Hardy–Weinberg Equilibrium; MAF, Minor Allele Frequency; PIHAT, proportion of identity-by-descent. Created with draw.io v16.2.7 (https://github.com/jgraph/drawio).

Samples from a total of 1024 donors were selected and utilized for SNP array genotyping. After QC filters based on the obtained genotypes (Fig. 1), we identified 863 unrelated individuals (406 males, 457 females) and 514,561 variants for further assessments. We also identified samples where, albeit all grandparents were born in the Canaries, they were not from the same island. The data from these samples was excluded from the analyses described in this study, although they will be considered in further development of the CIRdb catalog. The distribution of samples per island that will be considered in the analyses is as follows: 105 from El Hierro, 93 from La Palma, 141 from La Gomera, 156 from Tenerife, 210 from Gran Canaria, 47 from Fuerteventura, and 111 from Lanzarote.

### Population characteristics based on SNP arrays included in CIRdb catalog

**Figure 2:**
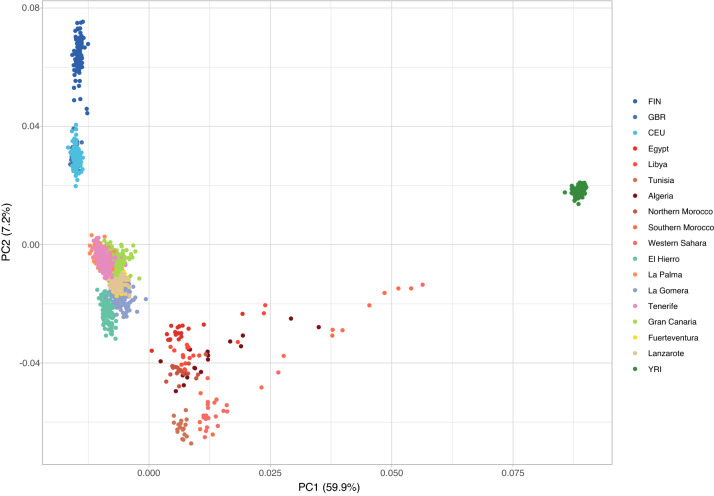
Representation of the first two principal components comprising 67.1% of genetic variation in Canary Islanders and reference populations from Europe (GBR, FIN, and CEU), North Africa (Algeria, Egypt, Libya, Northern Morocco, Southern Morocco, Western Sahara, and Tunisia), and Sub-Saharan Africa (YRI). A total of 101,271 variants and 1385 samples were used. Created with R v3.2.2 (https://www.r-project.org/).

In the PCA, including Canary Islanders and the reference populations (101,271 variants, 1385 samples), the first two principal components (PCs) encompassed 67.1% of the variation and revealed a distinctive separation in four main clusters (Fig. 2). Similar to what has been described recently by us^13^, the EUR and SSA individuals dominate the PC1 axis of differentiation, forming compact and well-separated clusters, also revealing a scattering pattern of clustering of NAF individuals from diverse populations. PC2 portrays the differentiation between NAF and EUR. In this axis of differentiation, the samples from the different Canary Islands clustered tightly between each other, but separately from the EUR, NAF, and SSA populations. In the cluster, within-Archipelago affinities are somehow evident, with some island populations plotting closer to NAF (El Hierro, La Gomera, Fuerteventura, and Lanzarote) while others situated closer to EUR (La Palma, Gran Canaria, and Tenerife).

**Figure 3:**
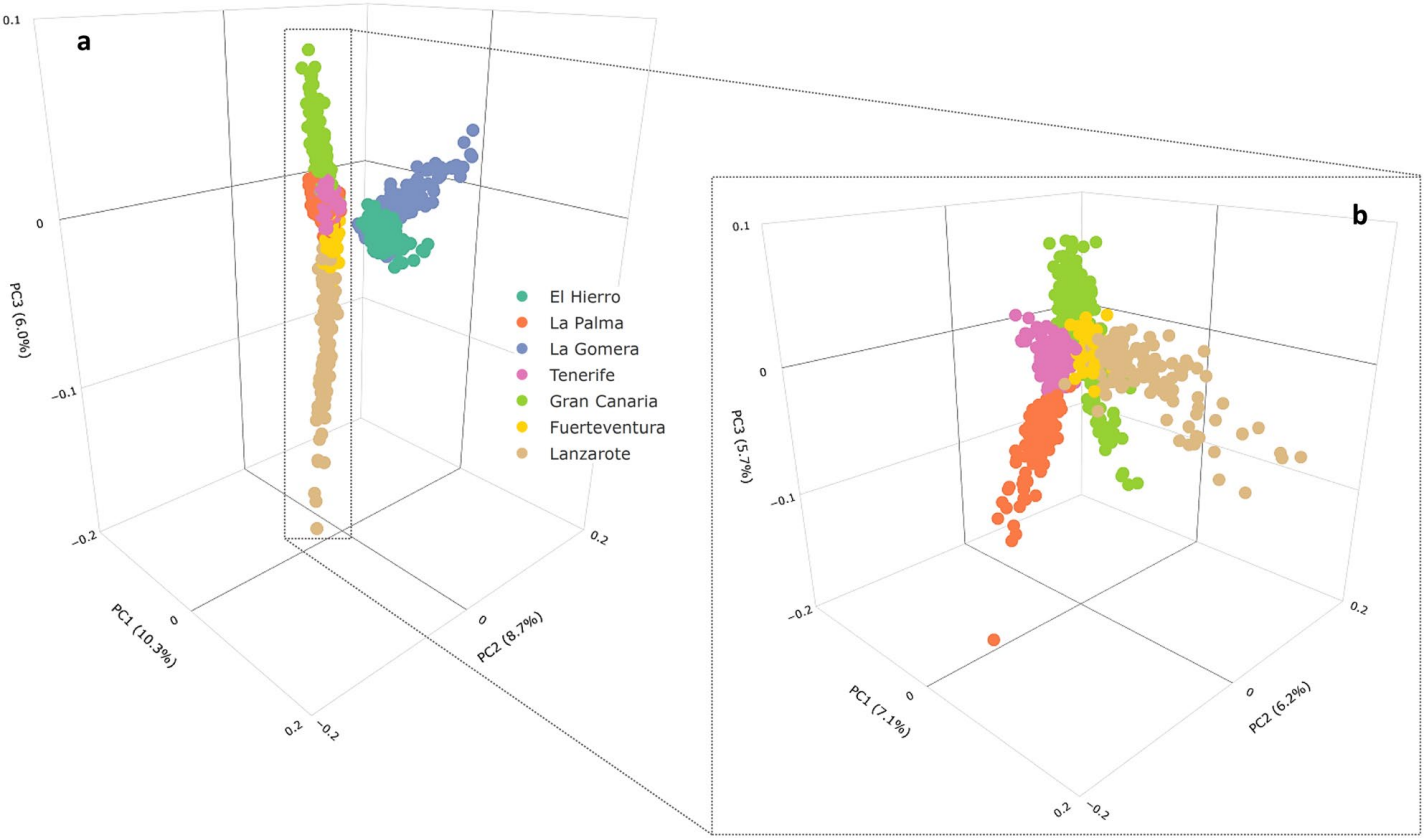
Representation of the first three principal components from PCA of Canary Islanders (a total of 116,959 variants were used). a) Including all unrelated Canary Islands samples (*N* = 863), where the first three PCs explain 25.0% of variability. b) Excluding the samples from El Hierro and La Gomera (*N* = 617), where the first three PCs explain 19.0% of variability. Created with R v3.2.2 (https://www.r-project.org/).

We also assessed the 863 unrelated Canary Islands donors (Fig. 3A) (116,959 variants, 863 samples) by PCA, where the first two PCs clearly distinguished the donors from El Hierro and La Gomera, the two smallest islands, from the rest of the islands. The rest of the island populations followed a continuum in the PC3 axis of differentiation, with a tendency to locate the samples from Gran Canaria, La Palma, and Tenerife on one side, and those from Fuerteventura and Lanzarote on the other. This differentiation is more evident when excluding the samples from El Hierro and La Gomera from the PCA analysis (Fig. 3B).

### Represented genetic ancestries in the CIRdb catalog

**Figure 4:**
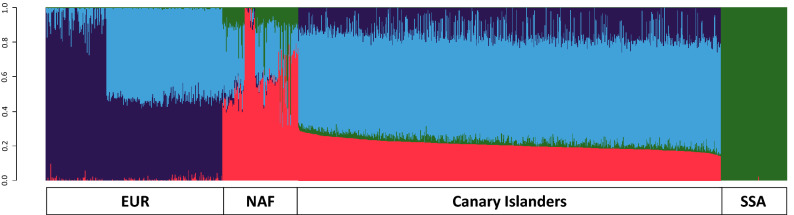
ADMIXTURE estimates for the best fitting model (*K* = 4) for the Canary Islanders and the reference populations. EUR, Europeans; NAF, North Africans; SSA, sub-Saharan Africans. A total of 1212 samples and 101,271 variants were used. Colors represent ancestry components aligned with different populations (dark blue, Northwestern Europe; light blue, Southeastern Europe; pink, North Africa; green, sub-Saharan Africa). Created with R v3.2.2 (https://www.r-project.org/).

ADMIXTURE ancestries for the dataset indicated that the best fitting model was obtained for *K* = 4 (Fig. 4). This is in agreement with previous results^11,12^ assessing the best fitting based on badMIXTURE residuals which ensured that *K* = 4 provided robust ancestry proportions^13^. Note, however, that the two EUR ancestries were aggregated into one for the rest of assessments to avoid relying on unstable subcontinental ancestry estimates given the small number of variants (Table 2). By aligning the identified clusters with the most abundant components identified in the references, they supported that the largest contribution to the genetic background of Canary Islanders is, on average, EUR ancestry (76.4%; composed of two ancestries aligned with the European northwest–southeast axis of differentiation^69^), followed by NAF (20.8%), and SSA (2.8%) (Table 2). Alternative analysis including also European populations from the south of the continent (TSI and IBS from 1KGP) to have information from the main European axis of differentiation barely changed the overall results (see Supplementary Fig. S1, Supplementary Table S1, and Supplementary Table S2 online). ELAI ancestries provided a similar scenario of admixture composed mainly by EUR (71.4%), followed by NAF (26.7%), and SSA (1.9%) (Table 2). However, we observed a much wider interindividual variation in the admixture proportions in Canary Islanders in this study with more samples from the geography (Fig. 4), so that the NAF and SSA ancestry assignations in Canary Islanders could be as high as 38.2% and 9.5%, respectively.

**Table 2:** Percentage of genomic ancestry proportions obtained by ADMIXTURE (*K* = 4, samples = 1212, variants = 101,271) and ELAI (14 generations, samples = 1212, variants = 114,929) in the Canary Islanders.

Ancestry (%)	ADMIXTURE			ELAI		
	Min	Average	Max	Min	Average	Max
EUR	66.5	76.4 ± 5.7*	84.9	59.4	71.4 ± 4.9	84.5
NAF	14.3	20.8 ± 3.0	30.6	15.0	26.7 ± 4.6	38.2
SSA	0.0	2.8 ± 1.6	9.5	0.0	1.9 ± 1.3	8.3

EUR, European; NAF, North African; SSA, sub-Saharan African. For Average columns, numbers refer to average ± standard deviation (in percentage). *European ancestry represents the sum of percentages from both Northwestern and Southeastern components.

When island populations were considered individually, the largest average NAF ancestries were obtained for El Hierro, La Gomera, Fuerteventura, and Lanzarote for both admixture estimators. La Gomera was also the island population with the largest SSA proportion on average (Table 3).

**Table 3:** Mean ancestry proportions obtained with ADMIXTURE (*K* = 4, samples = 1212, variants = 101,271) and ELAI (14 generations, samples = 1212, variants = 114,929) per island population.

Canary Islands	ADMIXTURE			ELAI		
	EUR*	NAF	SSA	EUR	NAF	SSA
El Hierro	77.7 ± 2.9	20.0 ± 2.1	2.3 ± 0.7	68.3 ± 2.6	31.0 ± 2.6	0.7 ± 0.4
La Palma	79.7 ± 2.6	18.8 ± 1.9	1.5 ± 0.8	76.5 ± 2.8	22.4 ± 2.7	1.1 ± 0.6
La Gomera	73.7 ± 2.9	21.6 ± 2.3	4.8 ± 1.3	65.8 ± 2.4	31.0 ± 2.6	3.2 ± 1.2
Tenerife	78.7 ± 2.4	19.7 ± 2.0	1.6 ± 0.9	75.3 ± 2.8	23.6 ± 2.6	1.1 ± 0.6
Gran Canaria	77.4 ± 2.5	19.3 ± 2.1	3.3 ± 1.5	73.7 ± 3.1	23.6 ± 2.5	2.7 ± 1.3
Fuerteventura	72.6 ± 2.8	24.6 ± 2.1	2.9 ± 1.1	67.2 ± 3.1	31.1 ± 2.8	1.7 ± 0.8
Lanzarote	72.3 ± 2.5	24.7 ± 2.4	3.1 ± 1.0	67.1 ± 2.8	30.9 ± 2.6	2.0 ± 0.7

EUR, European; NAF, North African; SSA, sub-Saharan African. All numbers refer to average ± standard deviation (in percentage). *European ancestry represents the sum of percentages from both Northwestern and Southeastern components.

### CIRdb: the first step for cataloging the natural genetic variation of the Canary Islands

**Figure 5:**
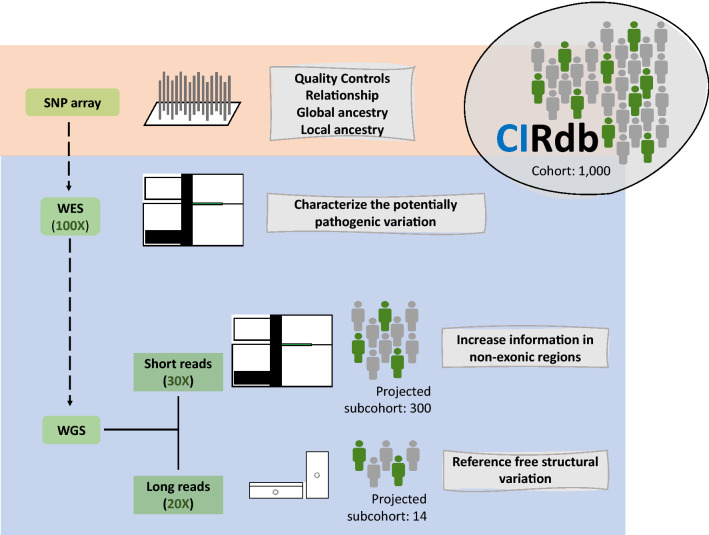
Overall schematic representation of the technologies and sample estimates projected for developing the catalog of natural genetic variation in the Canary Islanders. The sections corresponding to the data presented in this study (orange) and the work in progress (blue) are shown. CIRdb, Canary Islands Reference database; WES, whole-exome sequencing; WGS, whole-genome sequencing.

Considering SNP array data analyses as the starting point, the design for the reference genetic catalog of the Canary Islands population (CIRdb) is presented here for the first time. This catalog will be based on all the unrelated individuals identified in this study (irrespective of whether they declared that the four grandparents were born in the same island) and has been envisaged as a combination of data from three different technologies where each one provides its advantages for the genetic characterization of Canary Islanders. The conceptual design of CIRdb is shown in Fig. 5 and will involve the use of SNP array data (this study), as well as whole-exome, and whole-genome sequencing studies. In this regard, in-house bioinformatic pipelines for detecting single nucleotide variants, small insertions and deletions, and structural variants in whole-exome and whole-genome data are being developed and benchmarked against Genome In a Bottle standard materials^70^. Laboratory intercomparisons and updates are deposited in a publicly available repository (https://github.com/genomicsITER/benchmarking).

## Discussion

This study provides the largest genomic study of current Canary Islanders conducted to date, revealing unique population features and particular ancestry patterns based on SNP array data. In line with our previous studies with fewer samples^13^, we identify genetic peculiarities that differentiate the current Canary Islands populations from mainland populations^8–12^. Besides, with a larger sample size, we now evidence a clear pattern of genetic differentiation among islands not observed previously, where donors from El Hierro, La Gomera, Fuerteventura, and Lanzarote exhibited the largest average ancestry that can be assigned to NAF. We also evidenced the existence of more extreme individual NAF ancestries in the population (i.e., 38.2%) compared to previous estimates. An important isolation pattern of the populations from El Hierro and La Gomera, and the existence of further substructuring in the Canaries was also evident in this study. Taken together, this study evidenced the unique admixed makeup of current Canary Islanders and thus, establish the grounds for developing a catalog of genetic variation for this population that will be useful for the transition to Precision Medicine in the region.

There have been significant advances in Precision Medicine. From Archibald Garrod^71^ and the precursors of Precision Medicine, through one of the first examples of prevention, detection, and treatment of diseases tailored to individual profiles based on pharmacokinetics (i.e., warfarin)^72^, nowadays this paradigm encompasses diverse areas such as epigenetics, environmental exposures, imaging and radiology, and genetics and genomics, among others^73,74^. In this context, Genomic Medicine has emerged as a key discipline that has demonstrated important benefits in oncology^75^, pharmacology^76^, and rare and undiagnosed diseases^77^, to name a few, and including the possibility to improve the turnaround time, and in reducing the costs and the uncertainty of the diagnostic odyssey of the patients and their relatives^78,79^. In this context, many countries have seen the benefits of the pioneering implementation of genomic medicine within their healthcare system^80–82^. The first successful use of whole-exome sequencing to identify a disease-causing genetic mutation was reported about ten years ago, by Worthey in 2011^83^. A 15-month-old child was diagnosed with presumptive Crohn’s disease and treated accordingly without improvements in symptoms. After several years of diagnostic odyssey, a WES analysis identified a novel, hemizygous missense mutation in the X-linked inhibitor of apoptosis. This landmark study was followed by others based on the same concept and techniques but considering more patients and controls, sometimes without a clear clinical diagnosis in place before the analysis^78,84–88^. For whole-genome sequencing, several fruitful studies have also been carried out^28,89–91^. Nowadays, the routine implementation of NGS in clinical settings has drastically improved the average diagnostic yield from 10 to 36% (WES) or 41% (WGS), and the rate of clinical utility from 6 to 17% (WES) or 27% (WGS). Based on these benefits, many countries have extended these studies to comprise global, population-scale analyses including population controls, so that the natural genetic variation of the population could be also deeply characterized. In some cases, population classification is not entirely accurate and a more fine-scale analysis, based on ancestry, is needed^92^.

Following on this idea, here we present the study sample for the establishment of a reference genetic catalog for the current Canary Islanders. As the greatest fraction of rare genetic variation, which accumulates the most clinically relevant genetic variation, would remain understudied unless NGS technologies are in place, with CIRdb we envisage the use of a combination of several technologies to efficiently develop the population-specific catalog. As a starting point, we have assessed all samples to be included with the Axiom® Genome-Wide Human CEU 1 array as a first stage to efficiently characterize the global and local ancestry components, local substructure and inbreeding patterns, but also for the detection of samples that could be difficult to sequence or that had unknown family relationships with others in the cohort, allowing us to prioritize the samples for more expensive ulterior approaches. Considering the next steps, CIRdb plans to run WES in all the prioritized samples to efficiently examine the fraction of the genome that includes ~ 85% of all described disease-causing variants^93^. Using WES at population scale, it has been possible to detect an enrichment of risk variants for Panic Disorder in the Faroese population^94^, specific genetic loci associated with longevity in Bulgarian centenarians^95^, or study the metabolic impact of candidate effector genes in Southwestern American Indian population^96^, to name a few. WGS theoretically targets the entire DNA sequence of donors, offering the optimal solution for unbiased genetic studies although at higher costs per sample. Because of that, the use of WGS is projected in CIRdb as a complementary approach that will be used in subsets of the samples to improve the catalog and allowing to improve the imputation of genetic variation^97^ in the biomedical studies conducted in the Canary Islanders, as has been evidenced in Estonian and Native Hawaiian populations^18,38^. CIRdb aims to leverage two technologies for WGS, namely short-read sequencing (SRS) (Illumina, San Diego, CA, USA) and long-read sequencing (LRS) (Oxford Nanopore Technologies, Oxford, UK). The former will allow us to enrich the catalog with genetic information beyond the exome regions with high accuracy while containing the project costs. The latter will specifically enable the analysis of other types of genetic variation (e.g. structural variants, SVs)^98,99^, particularly beneficial for medically-relevant genes^100^ and assess the benefits of de novo assembly of genomes to assist in improving the population^101,102^. Studies in patients with Bardet-Biedl syndrome^103^ or Carney complex^104^ have shown the benefits of using LRS which would still be unsolved otherwise using SRS technologies.

Despite the forthcoming studies to build CIRdb will deepen in the genetic characterization of this population, we recognize some major issues of the study. Firstly, the number of evaluated SNPs (up to 114,929 in total in comparative studies) and a focus on autosomal variation limited our ability to assess the existent subcontinental influences^63,105^ in the ancestry analyses. This is the main reason for us to focus on the three continental ancestry components following our previous observations^13^. Forthcoming studies incorporating a much higher number of variants and the analysis of maternal (mtDNA) or paternal (NRY) lineages will be optimal to assess the subcontinental components of the admixture. Secondly, although relying on SNP arrays benefits from standardized pipelines and highly reproducible and reliable genotyping data, one of the most pronounced drawbacks of the study is the focus on one type of genetic variation (i.e., SNPs) and on alleles in the higher end of frequency spectrum. Information from structural and rare variation will provide new clues for disentangling the recent evolutionary history of this population and identify novel genetic links with disease. Filling these gaps will be the aim of leveraging different sequencing technologies for the establishment of the CIRdb catalog.

In summary, here we deepen into the genetic characterization of current Canary Islanders and establish the grounds for developing CIRdb to put forward a catalog of genetic variation for this population. CIRdb will be developed with complementary technologies and the tools and resources are currently under active development to create a precise public and available database for researchers and healthcare professionals.

## Supplementary Information

Supplementary Information.

## Data Availability

The data generated as part of this study has been deposited in the European Genome-Phenome Archive (EGA, https://ega-archive.org/studies/EGAS00001006050).
