# Supplementary material for: Developing CIRdb as a catalog of natural genetic variation in the Canary Islanders
Source: Sci Rep. 2022 Sep 27;12:16132. doi: 10.1038/s41598-022-20442-x (PMC9514705; doi:10.1038/s41598-022-20442-x)
Supplement: Supplementary file 1 — Supplementary Information. [file 41598_2022_20442_MOESM1_ESM.pdf]

Supplementary Information

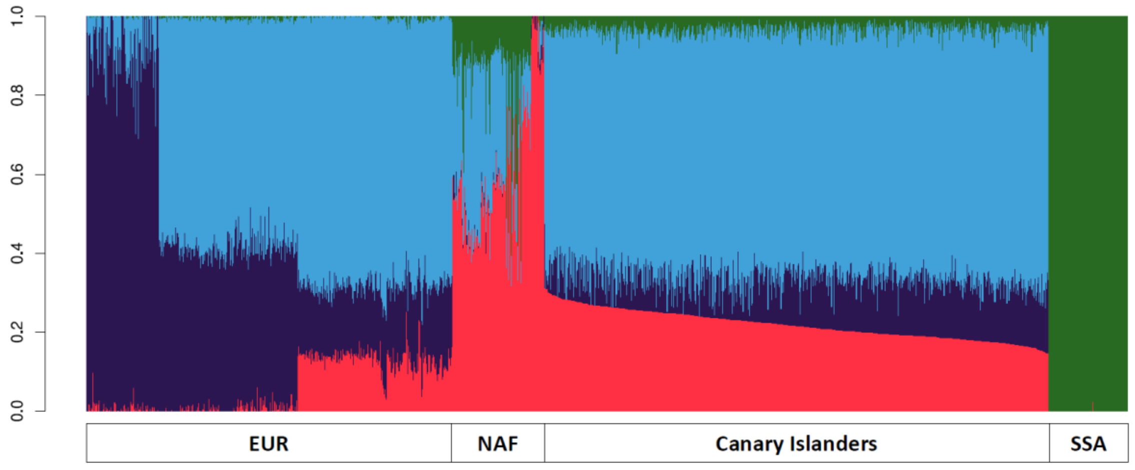

Supplementary Figure S1. ADMIXTURE estimates for the best fitting model (K=4) for the Canary Islanders and the reference populations. EUR, Europeans (from left to right: FIN, GBR, CEU, TSI, and IBS); NAF, North Africans; SSA, sub-Saharan Africans. Colors represent genomic components (dark blue, North Europe; light blue, South Europe; pink, North Africa; green, sub-Saharan Africa).

Supplementary Table S1. Pearson's product-moment correlation (R) between ancestry estimates from the experiment that did not include Toscani (TSI) or Iberian populations (IBS) *vs.* the experiment including them. EUR, Europeans; NAF, North Africans; SSA, sub-Saharan Africans. CI, confidence interval.

	p-value	95% CI	R
**North-EUR**	$< 2.2 \times 10^{-16}$	0.985 - 0.989	0.987
**South-EUR**	$< 2.2 \times 10^{-16}$	0.816 - 0.861	0.840
**NAF**	$< 2.2 \times 10^{-16}$	0.843 - 0.881	0.863
**SSA**	$< 2.2 \times 10^{-16}$	0.976 - 0.982	0.979

Supplementary Table S2. Mean ancestry proportions obtained with ADMIXTURE per island population and overall, when the experiments included Toscani (TSI) and Iberian populations (IBS) as part of the European reference population dataset.

	EUR	NAF	SSA
**Canary Islands**	74.8 ± 4.0	21.9 ± 3.7	3.3 ± 1.5
**El Hierro**	72.5 ± 2.6	25.4 ± 2.1	2.1 ± 0.6
**La Palma**	78.9 ± 2.5	19.0 ± 2.0	2.1 ± 0.8
**La Gomera**	70.2 ± 2.7	25.0 ± 2.5	4.8 ± 1.3
**Tenerife**	77.9 ± 2.4	19.9 ± 2.1	2.2 ± 0.9
**Gran Canaria**	76.8 ± 2.5	19.2 ± 2.2	4.0 ± 1.5
**Fuerteventura**	71.4 ± 2.8	25.2 ± 2.1	3.4 ± 1.1
**Lanzarote**	71.0 ± 2.5	25.4 ± 2.4	3.6 ± 1.0

EUR, European; NAF, North African; SSA, sub-Saharan African. Numbers refer to average ± standard deviation (in percentage).
